# Reporting guidelines for implementation and operational research

**DOI:** 10.2471/BLT.15.167585

**Published:** 2015-12-02

**Authors:** Simon Hales, Ana Lesher-Trevino, Nathan Ford, Dermot Maher, Andrew Ramsay, Nhan Tran

**Affiliations:** a Department of Strategy, Publications and Information, World Health Organization, avenue Appia 20, 1211 Geneva 27, Switzerland.; b Department of HIV, World Health Organization, Geneva, Switzerland.; c Special Programme for Research and Training in Tropical Diseases, World Health Organization, Geneva, Switzerland.; d Alliance for Health Policy & Systems Research, World Health Organization, Geneva, Switzerland.

## Abstract

In public health, implementation research is done to improve access to interventions that have been shown to work but have not reached many of the people who could benefit from them. Researchers identify practical problems facing public health programmes and aim to find solutions that improve health outcomes. In operational research, routinely-collected programme data are used to uncover ways of delivering more effective, efficient and equitable health care. As implementation research can address many types of questions, many research designs may be appropriate. Existing reporting guidelines partially cover the methods used in implementation and operational research, so we ran a consultation through the World Health Organization (WHO), the Alliance for Health Policy & Systems Research (AHPSR) and the Special Programme for Research and Training in Tropical Diseases (TDR) and developed guidelines to facilitate the funding, conduct, review and publishing of such studies. Our intention is to provide a practical reference for funders, researchers, policymakers, implementers, reviewers and editors working with implementation and operational research. This is an evolving field, so we plan to monitor the use of these guidelines and develop future versions as required.

## Résumé

Dans le domaine de la santé publique, des recherches sur la mise en œuvre sont menées pour améliorer l'accès aux interventions qui se sont révélées efficaces, mais qui n'ont pas touché toutes les personnes qui auraient pu en bénéficier. Les chercheurs identifient les difficultés pratiques qui compromettent les programmes de santé publique et s'efforcent de trouver des solutions pour améliorer les résultats sanitaires. Les données de programme systématiquement collectées dans le cadre des recherches opérationnelles, sont utilisées pour mettre en lumière des moyens de rendre les soins de santé plus efficaces, efficients et équitables. D'autre part, comme il est possible que les recherches sur la mise en œuvre portent sur de nombreux types de questions, différents plans de recherche peuvent s'avérer appropriés. Les directives existantes concernant l'établissement de rapports traitent en partie des méthodes utilisées dans le cadre des recherches sur la mise en œuvre et des recherches opérationnelles. Nous avons donc mené une consultation au sein de l'Organisation mondiale de la Santé (OMS), de l'Alliance pour la recherche sur les politiques et les systèmes de santé (AHPSR) et du Programme spécial de recherche et de formation concernant les maladies tropicales (TDR) et élaboré des directives pour faciliter le financement, la conduite, la révision et la publication de ce type de recherches. Notre objectif est de fournir une référence pratique pour les bailleurs de fonds, les chercheurs, les décideurs, les responsables de la mise en œuvre, les réviseurs et les éditeurs associés aux recherches sur la mise en œuvre et aux recherches opérationnelles. Ce domaine étant en constante évolution, nous prévoyons de suivre l'utilisation de ces directives et de rédiger, si besoin est, de futures versions.

## Resumen

En la salud pública, las investigaciones sobre la ejecución se llevan a cabo para mejorar el acceso a las intervenciones que se ha demostrado que funcionan pero que no han llegado a una gran parte de las personas que podrían beneficiarse de ellas. Los investigadores identifican los problemas prácticos a los que se enfrentan los programas de salud pública y tratan de encontrar soluciones que mejoren los resultados sanitarios. En las investigaciones operativas, se utilizan datos de programas recopilados rutinariamente para descubrir formas de ofrecer una atención sanitaria más efectiva, eficiente y equitativa. Puesto que una investigación sobre la ejecución puede abordar muchos tipos de cuestiones, pueden ser apropiados muchos diseños de investigación. Las directrices existentes sobre la presentación de informes cubren parcialmente los métodos utilizados en las investigaciones operativas y sobre la ejecución, por lo que se llevó a cabo una consulta a través de la Organización Mundial de la Salud (OMS), la Alianza para la Investigación en Políticas y Sistemas de Salud (Alianza IPSS) y el Programa Especial de Investigaciones y Enseñanzas sobre Enfermedades Tropicales (TDR) y se desarrollaron directrices para facilitar la financiación, realización, revisión y publicación de dichos estudios. El objetivo es proporcionar una referencia práctica para financiadores, investigadores, responsables de la formulación de políticas, implementadores, revisores y editores que trabajen con investigaciones operativas y sobre la ejecución. Se trata de un área en evolución, por lo que prevemos supervisar el uso de estas directrices y desarrollar versiones futuras si fuera necesario.

## ملخص

يتم إجراء البحوث التنفيذية في مجال الصحة العمومية لتطوير سبل الوصول إلى برامج التدخل العلاجي، والتي ثبت فعاليتها، لكنها لم تصل إلى العديد من الأفراد ممكن يمكنهم الاستفادة منها. ويحدد الباحثون المشكلات العملية التي تواجه برامج الصحة العمومية، كما يهدفون إلى إيجاد الحلول التي تحسّن من المحصلات الصحية. أما في مجال البحوث الميدانية، فإنه يتم توظيف البيانات الخاصة بالبرامج التي يتم جمعها بشكل روتيني للكشف عن سبل تقديم رعاية صحية تراعي عوامل الكفاءة والفعالية والإنصاف. وبما أنه يمكن للبحوث التنفيذية تناول أنواعًا عديدة من القضايا، فقد يكون من المناسب الاعتماد على العديد من تصاميم البحوث. ونظرًا لأن المبادئ التوجيهية الحالية لرفع التقارير لا تغطي سوى جانب من الوسائل المستخدمة في البحوث التنفيذية والميدانية، فقد طلبنا الاستشارة من خلال منظمة الصحة العالمية، واتحاد سياسات وبحوث النظم الصحية (AHPSR)، والبرنامج التخصصي للبحوث والتدريب المتعلق بالأمراض الاستوائية (TDR)، والمبادئ التوجيهية الموضوعة لتيسير جهود تمويل تلك الدراسات وإجرائها ومراجعتها ونشرها .يتمثل غرضنا في تقديم مرجع عملي للممولين والباحثين وصناع السياسات والقائمين على التنفيذ والمراجعة والمحررين المعنيين بالتعامل مع البحوث التنفيذية والميدانية. ****وبما أن هذا المجال آخذ في التطور، فإننا نخطط لمراقبة توظيف هذه المبادئ التوجيهية ووضع رؤى مستقبلية حسب الحاجة.

## 摘要

公共卫生领域中开展了实施性研究，旨在改善干预措施的普及率，这已表明发挥一定的作用，但是尚未普及到许多本应从中获益的人群。 研究人员确定公共卫生项目所面临的实际问题并且努力寻找改善卫生状况的解决方案。 在操作性研究中，我们使用定期收集的项目数据来揭示提供更加有效、高效和公平的医疗保健的途径。 由于实施性研究可以解决许多类型的问题，因而多种研究设计可能都是适合的。现存的报告指南部分涵盖了实施性和操作性研究中所采用的方法，因此我们通过世界卫生组织 (WHO)、卫生政策与卫生体制研究联盟 (AHPSR)、热带疾病研究特别行动规划 (TDR) 开展了一项咨询，并且制定了指南，旨在为此类研究提供经费、实施、评审和发表支持。我们的目的是为实施性和操作性研究的资助者、研究人员、政策制定者、执行者、评审者和编辑人员提供切实可行的参考。 这是一个不断发展的领域，因此我们计划监测这些指南的使用情况并根据需要制定出新的版本。

## Резюме

Исследования внедрения в здравоохранении проводятся для того, чтобы увеличить доступность тех вмешательств, которые хорошо себя зарекомендовали, но недоступны для многих людей, которым они могли бы быть полезны. Исследователи выявляют практические проблемы, с которыми сталкиваются программы общественного здравоохранения, и преследуют цель обнаружения решений, которые улучшают результаты лечения. В ходе оперативных исследований регулярно собираемые данные о программе используются для того, чтобы раскрыть способы создания более эффективного, действенного и общедоступного здравоохранения. Так как исследования внедрения могут быть посвящены разнообразным вопросам, существует множество приемлемых вариантов построения такого исследования. Существующие рекомендации по составлению отчетности лишь частично охватывают методы, используемые в ходе исследований внедрения и оперативных исследований, поэтому мы провели консультации со Всемирной организацией здравоохранения (ВОЗ), Союзом исследований стратегий и систем здравоохранения (AHPSR) и Специальной программой по научным исследованиям и подготовке специалистов в области тропических болезней (TDR) и разработали рекомендации для содействия финансированию, проведению, анализу и публикации таких исследований. Нашей целью является создание практического справочника для тех, кто финансирует исследования, проводит их, разрабатывает стратегии, занимается осуществлением, анализом и издательской работой в области исследований внедрения и оперативных исследований. Это поле деятельности пока только находится в процессе становления, поэтому мы планируем отслеживать использование данных рекомендаций и будем разрабатывать новые версии по мере необходимости.

## Introduction

Implementation and operational research are growing in importance and recognition. Major donors, including the Canadian Institutes of Health Research, the European & Developing Countries Clinical Trials Partnership, the United States of America’s National Institutes of Health and the Wellcome Trust are increasing funding support for this research area and leading scientific journals have established sections promoting the publication of such research. Implementation research contributes a growing part of the evidence base used by the World Health Organization (WHO), which promotes, supports, publishes and evaluates such research.

The combined results of basic, clinical and implementation research have made it possible to reach millions of people with life-saving treatments and effective prevention measures. For example, the global scale-up of antiretroviral drugs for human immunodeficiency virus (HIV) has brought the number of people on treatment to 13.6 million from 800 000 in 2003^1^ and reduced deaths from 2.4 million in 2005 to 1.5 million in 2013.^2^ As another example, tuberculosis is declining rapidly. However, to sustain these gains and reach elimination targets for tuberculosis, new drugs and vaccines are needed, along with the implementation research that shows how these can be delivered where they are needed and in a form that works. Deaths from malaria have decreased by 58% between 2000 and 2015,^1^ in part because malaria control measures, including indoor residual spraying, long-lasting insecticidal nets, point-of-care diagnostic tests and artemisinin-based combination therapies have been delivered effectively to communities.

Operational research uses an existing resource – the data routinely collected by programmes – to provide ways of improving programme operations and thereby delivering more effective, efficient and equitable care. Implementation and operational research are usually carried out in close collaboration between researchers and public health practitioners. Operational research is typically very specific to a single programme or activity. The costs are generally modest, yet these studies have the potential for a huge magnifier effect, in extending the impact of health interventions.^3^

Implementation research can help answer questions about why effective interventions are not reaching the people who could benefit from them.^4^^,^^5^ Implementation research is also useful in understanding how health system failures create barriers to the delivery of policies or programmes. For example, the non-adherence of providers to service delivery guidelines based on evidence may result from a lack of monitoring and feedback mechanisms within the system. It could also result from the time allocated per visit which limits what providers are able to achieve. A broad understanding of systems failures and their relationship to implementation barriers is a key aspect of much implementation research. Resolving barriers such as non-adherence to treatment guidelines may have less to do with training providers and more to do with changing the system to allow more time or by establishing better feedback mechanisms.

As the range of applications of implementation research is very broad, a wide range of different research methods may be used depending on the type of problem studied (Table 1). Existing guidelines and their extensions cover some – but not all – of the required reporting areas. These gaps can make it difficult for researchers, implementers and journal editors to ensure that studies are reported in sufficient detail to allow replication. A further difficulty is that the success of implementation, particularly for complex interventions, is often highly dependent on the context. The traditional structure of a scientific research article may not provide a good framework for reporting important contextual issues.

**Table 1:** Research objectives, implementation questions and research methods^4^

Objective	Description	Implementation question	Research methods
Explore	Explore an idea or phenomenon to make hypotheses or generalizations from specific examples	What are the possible factors and agents responsible for good implementation of a health intervention? For enhancing or expanding a health intervention?	Qualitative methods: grounded theory, ethnography, phenomenology, case studies and narrative approaches; key informant interviews, focus groups, historical reviews
			Quantitative: network analysis, cross-sectional surveys
			Mixed methods: combining qualitative and quantitative methods
Describe	Identify and describe the phenomenon and its correlates or possible causes	What describes the context in which implementation occurs? What describes the main factors influencing implementation in a given context?	Quantitative: cross-sectional (descriptive) surveys, network analysis
			Qualitative methods: grounded theory, ethnography, phenomenology, case studies and narrative approaches; key informant interviews, focus groups, historical reviews
			Mixed methods: both qualitative and quantitative inquiry with convergence of data and analyses
Influence	Test whether an intervention produces an expected outcome		
With adequacy	With sufficient confidence that the intervention and outcomes are occurring	Is coverage of a health intervention changing among beneficiaries of the intervention?	Before-after or time series in intervention recipients only; participatory action research
With plausibility	With greater confidence that the outcome is due to the intervention	Is a health outcome plausibly due to the implemented intervention rather than other causes?	Concurrent, non-randomized cluster trials: health intervention implemented in some areas and not in others; before-after or cross-sectional study in programme recipients and non-recipients; typical quality improvement studies
With probability	With a high (calculated) probability that the outcome is due to the intervention	Is a health outcome due to implementation of the intervention?	Partially controlled trials: pragmatic and cluster randomized trials; health intervention implemented in some areas and not in others; effectiveness-implementation hybrids
Explain	Develop or expand a theory to explain the relation between concepts, the reasons for the occurrence of events, and how they occurred	How and why does implementation of the intervention lead to effects on health behaviour, services, or status in all its variations?	Mixed methods: both qualitative and quantitative inquiry with convergence of data and analyses
			Quantitative: repeated measures of context, actors, depth and breadth of implementation across subunits; network identification; can use designs for confirmatory inferences; effectiveness-implementation hybrids
			Qualitative methods: case studies, phenomenological and ethnographic approaches with key informant interviews, focus groups, historical reviews
			Participatory action research
Predict	Use prior knowledge or theories to forecast future events	What is the likely course of future implementation?	Quantitative: agent based modelling; simulation and forecasting modelling; data extrapolation and sensitivity analysis (trend analysis, econometric modelling)
			Qualitative: scenario building exercises; Delphi techniques from opinion leaders

Note: Table reproduced from Peters, et al. ^4^

For the past 10 years, the *Bulletin of the World Health Organization* has published many examples of implementation research in a section called Lessons from the Field.^6^ The papers in this section have described actions taken in response to a wide variety of public health problems in many different settings, but we have found that authors often needed to be prompted to provide sufficient detail on local context, details of interventions and measures of impact. 

Here we describe the development of reporting guidelines for implementation and operational research (Table 2). In future, authors submitting relevant research articles to the *Bulletin* will be asked to follow the new reporting guidelines. The guidelines are the result of collaboration between WHO, the Alliance for Health Policy & Systems Research (AHPSR) and the Special Programme for Research and Training in Tropical Diseases (TDR), journal editors, researchers and funders. Our intention is to provide a useful reference for all involved in implementation and operational research, and to revise these guidelines, as required, after a first year’s trial period.

**Table 2:** Reporting guidelines for operational/implementation research^a^

Section	Reporting item
**Title and abstract**	Identify as implementation or operational research in the title. Provide a structured summary of study context, rationale, objectives, design, methods, results and conclusions.
**Introduction**	
Background	Explain the scientific background relating to both the intervention and the implementation. What is already known about the issue? Describe the policy or programme context. Describe relevant elements of setting or settings (for example, geography, physical resources, organizational culture, history of change efforts). What is it about implementation in this setting that warrants research and reporting of findings?
Problem	Briefly describe the nature and severity of the specific issue or problem that was addressed. Specify who (champions/supporters) what (events/observations) triggered the decision to make changes, why in this location and why now?
Implementation strategy	Describe mechanisms or strategies by which components were expected to cause changes, and plans for testing whether these were effective.
Intervention	What evidence-based intervention or innovation is proposed?
Intended outcomes	Describe the specific aim of the proposed study (changes/improvements in processes and outcomes).
**Methods**	
Study design	Identify the study design (for example, observational, quasi-experimental, experimental, qualitative, mixed) chosen for measuring impact of the intervention on primary and secondary outcomes, (if relevant).
Setting	Exact details of study locations, baseline population characteristics, recruitment of participants, relevant dates for implementation, follow-up, and data collection.
Implementation	Give a description of the implementation strategy: frequency, duration, intensity, including how and when interventions were actually implemented, additional resources required to support implementation, mode of delivery, why and when the study ended.
	Describe the intervention, (if relevant). The amount of detail given should be sufficient to allow replication of the study. For well-established interventions, it is sufficient to refer to previously published studies.
	Explain methods used to assure data quality (for example, blinding; repeating measurements and data extraction; training in data collection; collection of sufficient baseline measurements).
Participants	For qualitative studies: what was the approach (e.g. ethnography, grounded theory, narrative) and theory? Indicate how size of target population was determined. *Cohort study *– Give the eligibility criteria, and the sources and methods of selection of participants. Describe methods of follow-up.
	*Case-control study *– Give the eligibility criteria, and the sources and methods of case ascertainment and control selection. Give the rationale for the choice of cases and controls.
	*Cross-sectional study* – Give the eligibility criteria, the sources and methods of selection of participants.
	For matched studies, give matching criteria and number of exposed and unexposed or the number of controls per case. For randomized studies, how was randomization done, definition of clusters for cluster randomized studies. Was the study blinded?
Variables	Clearly define all outcomes, exposures, predictors, potential confounders, and effect modifiers.
Data sources/measurement	For each variable of interest, give sources of data and methods of assessment (or measurement). Describe sampling strategies and comparability of assessment methods if there is more than one group. Methods for processing data before and during analysis, including translation, transcription, data entry, data management and security, verification of data integrity, data coding, and de-identification. Explain how variables were handled in the analyses. If applicable, describe which groupings were chosen and why; how data were coded.
Analyses	Which analyses were pre-specified, and which were exploratory? For qualitative analyses: process by which inferences or themes were identified and developed, including the researchers involved in data analysis. For quantitative analyses: describe statistical methods, including those used to adjust for sampling methods and control for confounding. Where both qualitative and quantitative analyses are used, describe both types of analysis and how findings were synthesized. Describe any methods used to examine subgroups and interactions. Explain how missing data were addressed. Cohort study: explain how loss to follow-up was addressed. Case-control study: describe matching of cases and controls.
Ethical considerations	Including consent procedures, if relevant. How was confidentiality ensured? How was the balance between the potential risks and benefits of this research to individuals or communities assessed?
**Results**	
Descriptive data	Report numbers of individuals at each stage of study – e.g. numbers eligible, included in the study, completing follow-up, and analysed. Include a flow diagram, timeline or graph, if relevant. Cross tabulate the number of participants by subgroups as relevant e.g. demographic, clinical, social characteristics, response rates, loss to follow-up or other sources of missing data, potential confounders, for those who receive the intervention and those who do not receive it.
Outcomes	Explain the actual course of the intervention, if relevant. For example, describe the sequence of steps, events or phases; type and number of participants at key points, preferably using a time-line diagram or flowchart.
	Document the degree of success in implementation: • changes in processes and outcomes associated with the intervention. • changes observed in outcome (for example, population behaviour change, morbidity, mortality, function, patient/staff satisfaction, service utilization, cost, care disparities). • consider benefits, harms, costs, unexpected results, problems, failures.
Outcome data	Report numbers of outcome events (or summary measures over time), separately for those who receive the intervention and those who do not receive it. Include summary statistics and measure of variance (SD or SE).
Main results	Main findings (e.g. interpretations, inferences, and themes); might include development of a theory or model, or integration with prior research or theory. Provide unadjusted estimates of intervention effect, and, if applicable, confounder-adjusted estimates and their precision (e.g. 95% confidence interval). Consider translating estimates of relative risk into absolute risk for a meaningful time period. Synthesis of quantitative and qualitative results.
Other analyses	Report other analyses done – e.g. analyses of subgroups and interactions, sensitivity analyses, costs.
**Discussion**	
Key results	Summarize key results with reference to study objectives.
Limitations	Discuss limitations of the study, taking into account possible sources of confounding, bias or imprecision in design, measurement, and analysis that might have affected study outcomes (internal validity). Discuss both direction and magnitude of any potential bias.
Interpretation	Interpret the results considering objectives, limitations, multiplicity of analyses, results from similar studies, and other relevant evidence. Compare and contrast study results with relevant findings of others, drawing on broad review of the literature; use of a summary table may be helpful in building on existing evidence. Suggest steps that might be modified to improve future performance. Review issues of opportunity cost and actual financial cost of the intervention.
Contextual factors	Success factors, barriers and how they were overcome.
Generalizability	Discuss the generalizability (external validity) of the study results. Explore factors that could affect generalizability – for example, representativeness of participants; effectiveness of implementation; dose–response effects; features of local setting. Applicability to other settings; Potential barriers to scale up.
**Conclusion**	Consider overall practical usefulness of the intervention. Suggest implications for the implementation programme. How will the results be used/translated into practice in the context of the study? Suggest implications for further studies.
**Other information**	Indicate if the study is registered and if the data are available.
	Give the source of funding and the role of the funders for the present study and, if applicable, for the original study on which the present article is based. State the role of individuals in the study and any conflict of interest.

SD: standard deviation; SE: standard error.

^a^ The reporting items in this table are intended to cover the wide range of study designs for implementation and operational research. As a result, not all of the items are relevant for all studies (for example, some studies will not involve the testing of an implementation strategy).

## Guidelines development

**Fig. 1:**
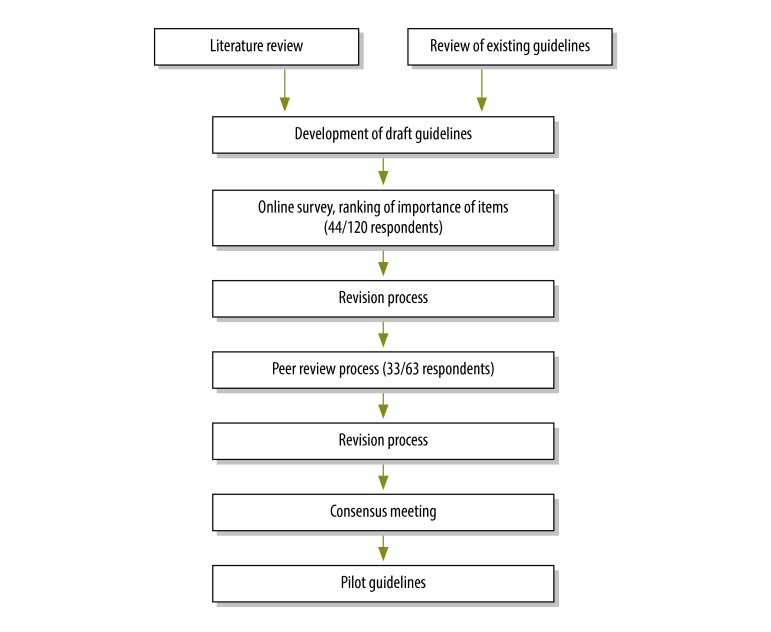
Flow chart illustrating the process of guideline development

A flowchart summarizing the development of the guidelines is shown in Fig. 1. We reviewed existing guidelines, and guidelines under development on the EQUATOR website^7^ and selected relevant items from a set of existing guidelines listed in Table 3. Since many of the items in the standards for reporting observational research (STROBE) guidelines^8^ were selected, we used the STROBE checklist as a starting point. We compiled a list of 120 researchers, funders, editors and implementers and sent them a link to an online survey, asking that they score the importance of potential reporting items. We received 44 responses from people working in a wide range of countries. Most respondents identified themselves as researchers.

**Table 3:** Reporting guidelines^a^

Type of study	Guideline name	Example extensions
Randomized trials	CONSORT	TIDieR
Observational studies	STROBE	RECORD
Systematic reviews	PRISMA	PRISMA-P
Qualitative research	SRQR	COREQ
Diagnostic/prognostic studies	STARD	TRIPOD
Quality improvement studies	SQUIRE	
Economic evaluations	CHEERS	
Phase IV implementation studies	STaRI	
Policy interventions	UNTIDieR	

^a^ Adapted from http://www.equator-network.org

Several respondents suggested including more items relevant to qualitative studies. Others pointed out that implementation studies often, but not always, involve defining a problem to be solved. In some cases, reasons for the success of a programme might be the main focus of a study. There was a range of opinions on the right balance between reporting of the implementation strategy as opposed to specific details of an intervention. Some expressed a concern that implementers may be put off rather than encouraged if the proposed guidelines were too detailed.

A revised version was sent for review by email and 33 responses were received. To gain consensus on key issues and help reconcile comments from people with different perspectives, a consensus meeting was held at WHO headquarters in October 2015. Participants agreed on the inclusion of a set of standard reporting items and pointed out that the guidelines should not refer exclusively to health-care settings. Some suggested that the guidelines should refer to existing guidelines where relevant. For example, studies using qualitative methods could use the SRQR guidelines;^9^ studies using routine data could use RECORD.^10^ In revising the guidelines, we tried to balance completeness with user-friendliness and decided that it was preferable to produce a comprehensive guideline, as shown in Table 2, that includes all relevant items, rather than referring authors to multiple guidelines.

## Conclusion

A major challenge in the development of reporting guidelines for implementation and operational research is that this research is governed by the nature of the questions rather than by specific methods or designs. As such, the guidelines presented in this paper build upon and bring together a range of existing guidelines. The process of developing these reporting guidelines has brought people with different expertise and perspectives to the debate and helped build consensus. It is hoped that the present guidelines will be a useful reference, but further discussion and development will be required to overcome challenges in this evolving field.
